# Green synthesis of zinc oxide nanoparticles from *Wodyetia bifurcata* fruit peel extract: multifaceted potential in wound healing, antimicrobial, antioxidant, and anticancer applications

**DOI:** 10.3389/fphar.2024.1435222

**Published:** 2024-08-05

**Authors:** Adel Moalwi, Keerti Kamat, Uday M. Muddapur, Bader Aldoah, Hajar Hassan AlWadai, Abdulrahman Manaa Alamri, Fauwaz Fahad Alrashid, Saeed Ali Alsareii, Mater H. Mahnashi, Ibrahim Ahmed Shaikh, Aejaz Abdullatif Khan, Sunil S. More

**Affiliations:** ^1^ Department of Surgery, College of Medicine, Najran University, Najran, Saudi Arabia; ^2^ Department of Biotechnology, BVB Campus, KLE Technological University, Hubballi, Karnataka, India; ^3^ Department of Surgery, College of Medicine, Hail University, Hail, Saudi Arabia; ^4^ Department of Pharmaceutical Chemistry, College of Pharmacy, King Khalid University, Abha, Saudi Arabia; ^5^ Department of Pharmacology, College of Pharmacy, Najran University, Najran, Saudi Arabia; ^6^ Department of General Science, Ibn Sina National College for Medical Studies, Jeddah, Saudi Arabia; ^7^ School of Basic and Applied Sciences, Dayananda Sagar University, Bangalore, India

**Keywords:** *W. bifurcata*, nanoparticles, antibacterial activity, wound healing, antioxidant activity

## Abstract

This study focuses on the synthesis, characterization, and use of zinc oxide nanoparticles (ZnONPs) derived from *W. bifurcata* fruit peel extract. ZnONPs are frequently synthesized utilizing a green technique that is both cost-effective and ecologically friendly. ZnONPs were characterized utilizing analytical techniques. Ultra Violet visible (UV-Vis) spectra showed peaks at 364 nm, confirming the production of ZnONPs. Scanning Electron Microscope analysis indicated that the nanoparticles generated were spherical/agglomerated, with diameters ranging from 11 to 25 nm. FTIR spectroscopy was used to identify the particular functional groups responsible for the nanoparticles’ reduction, stabilization, and capping. Phytochemical analysis of the extract revealed that flavonoids, saponins, steroids, triterpenoids, and resins were present. The antibacterial activity of *W. bifurcata* synthesised nanoparticles was evaluated against pathogenic bacteria. The ZnONPs antioxidant activity was assessed using DPPH assay. The *in vitro* cytotoxicity was assessed against prostate cancer PC3 cells. The wound healing potential was assessed by employing *in vitro* scratch assay and *in vivo* excision model in Wistar rats. Because of its environmentally benign production, low toxicity, and biocompatibility, ZnONPs exhibited potential antibacterial, antioxidant, anticancer, and wound healing activities, indicating that they could be used in cancer treatment and wound management. Further study is required to examine the fundamental mechanisms and evaluate the safety and effectiveness of the test sample in clinical situations.

## Graphical Abstract

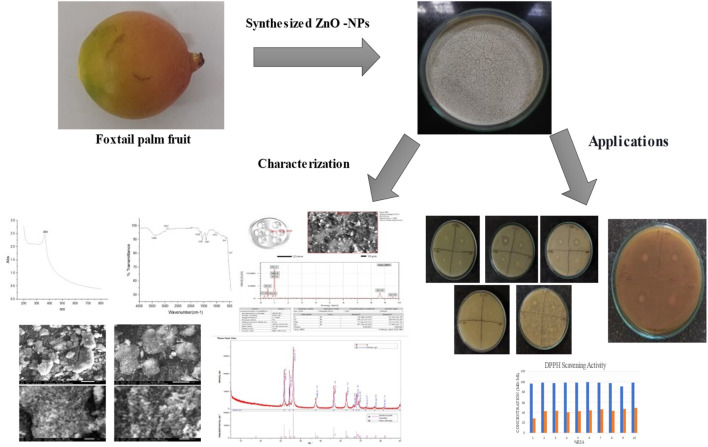

## 1 Introduction

Nanotechnology is an interdisciplinary field that focuses on the study and manipulation of materials at the nanoscale, typically in the range of 1–100 nm (Roco, 2003; Abbes et al., 2022). This emerging technology has found widespread applications in various industries, including pharmaceuticals, chemicals, and food processing (Faisal et al., 2021; Neamah et al., 2023). Green-synthesized nanoparticles have found diverse applications in the biomedical field, including drug delivery, tissue engineering, and wound healing, owing to their unique physicochemical properties and biocompatibility. These nanoparticles have demonstrated antimicrobial, antioxidant, and anti-inflammatory activities, making them promising candidates for the development of novel therapeutic and diagnostic strategies (Abdelmigid et al., 2022; Mahmood et al., 2022; Kadhim et al., 2023; Neamah et al., 2023; Mohammed et al., 2024). Moreover, the small size of nanoparticles allows them to readily interact with receptors, nucleic acids, and cell membranes, making them highly useful in the field of medicine (Hamrayev et al., 2021).

Zinc Oxide (ZnO) is an inorganic metal oxide that consists of nanostructures in a vast range (Parthasarathy et al., 2016). ZnO has a variety of unique qualities that set it apart from other materials, including low toxicity, a broad spectrum of radiation adsorption, high photo stability, high chemical stability, and high electrochemical coupling coefficient. Biocompatibility, biodegradable, and rigidity make ZnO an interesting material in biomedicine field. ZnO can adsorb UV-A and UV-B. Nowadays ZnO is used in many sunscreen lotions, ointments, creams, dental pastes, and medicines and it found that ZnO has wound-healing properties which are used in inflammation and itching (Manokari et al., 2016).

Nanoparticles fabrication is possible through physical, chemical, and green synthesis methods (Abbes et al., 2022). The eco-friendly synthesis of nanoparticles has proposed other traditional methods of synthesis of nanoparticles and this method uses plant extract and has been approved as an inexpensive way of synthesizing the nanoparticles (Manokari et al., 2016). Among metal oxides, Zinc oxide nanoparticles are considered safe, biocompatible, and non-toxic (Abdelmigid et al., 2022). Recently, ZnONPs have been synthesized by various methods like thermal decomposition, microwave irradiation, Sono chemical, and sol-gel (Raja Ahmad et al., 2019). Zinc oxide nanoparticles synthesized using plant extracts have wide biological properties such as anticancer, antimicrobial, antibacterial, and antifungal activities (Faisal et al., 2021). This nanoparticle is used in many industrial applications like cosmetics, sunscreen creams, and paints (Shaban et al., 2022). In comparison with other biological processes, the utilization of peel extracts for producing nanoparticles is advantageous as fruit peels are eco-friendly easy to obtain and affordable and have high bioactive compounds and these bioactive compounds have the potential to act as antioxidants and antimicrobials (Balavijayalakshmi and Ramalakshmi, 2017). A promising method of waste water treatment is offered by ZnO nanoparticles by Photocatalytic activity (Parthasarathy et al., 2016).

*W. bifurcata*, often known as foxtail palm fruit, and belongs to the family Arecaceae and is grown for landscaping purposes. The foxtail fruits are never specifically used; they are always left to perish (Nik Yusoff et al., 2022). Approximately 12 years after reaching maturity, foxtail palms can generate inflorescences. Large, round, green fruits that eventually become orange red are produced by fertilized female flowers. Approximately 1 _1/4_ inches broad are mature fruits. Each fruit is a single seed that is 2_1/4_ inches long (Perez et al., 2009).

The main focus of the study was to produce ZnO nanoparticles using the foxtail palm fruit peel extract, characterize the synthesized ZnONPs, optimization of various parameters like pH and temperature, and investigate biomedical applications like antimicrobial, antifungal, antioxidant, anticancer, and wound healing potential by employing different *in vitro* and *in vivo* models.

## 2 Methods and materials

### 2.1 Sample collection and preparation of extract

*W. bifurcata* was collected from the KLE Technological University campus Hubballi, Karnataka, India. The fruit was washed, and the peel was peeled, then 15 g of peel was weighed and after adding 150 mL of distilled water, it boiled for 15 min. The extract was allowed to rise to room temperature before being chilled for future use and filtered through Whatman’s filter paper.

### 2.2 Zinc nanoparticle synthesis

Zinc acetate solution (0.1 M) was prepared using 6.585 g zinc acetate and dissolved in 300 mL of distilled water which was kept in a magnetic stirrer for 1 h. After 1 h when zinc acetate is dissolved add 60 mL of extract dropwise to 240 mL of zinc acetate while the stirring should be continuous. After 1 h of adding extract pH was set to 10 by using 2 M freshly prepared NaOH. Then keep the solution in the water bath at 50°C for 1 h then again keep it in the stirrer for 2 h. A white crystalline precipitation was formed which was allowed to settle. After disposing of the excess fluid, the white crystalline precipitate was scraped off and preserved for later use after being hot-dried at 50°C in the oven.

### 2.3 Characterization of synthesized ZnONPs

UV-Vis spectroscopy, Fourier transform infrared spectrophotometry (FTIR), energy dispersive X-ray spectroscopy (EDS), and X-ray diffraction (XRD) were used to characterise the generated ZnONPs.

### 2.4 UV visible spectroscopy analysis of ZnONPs

The nanoparticle’s size has a significant impact on the overall characteristics of materials. To investigate the properties of the materials the size development of semiconducting nanoparticles becomes extremely important. A popular approach for analysing the optical characteristics of nanosized particles is UV-Vis absorption spectroscopy (Talam et al., 2012). To determine the stability and production of nanoparticles UV Spectroscopy was used (Ifeanyichukwu et al., 2020). ZnONPs powder obtained from the extract was mixed in deionised water and adsorption was taken at 300–600 nm by using a UV 9600A UV/Visible Spectrophotometer (Shimadzu Instruments Co., Ltd., Kyoto, Japan).

### 2.5 FTIR analysis of synthesized zinc oxide nanoparticles

FTIR spectra were produced after electromagnetic waves with a frequency range of 400–4,000 cm^-1^ were absorbed. A wide range of spectrum information is gathered by the FTIR spectrometer. By absorbing electromagnetic waves at specific frequencies and intensities, different functional groups, and chemical structures in nano scaled ZnO may be identified. As a result, the various groups and structures exhibit the usual band arrangement and shape for ZnO nanostructures (Manjunatha et al., 2019). Thermo Scientific Nicolet iSTM50 FTIR Spectrometer was used to conduct Fourier Transform Infrared Spectroscopic analysis.

### 2.6 Scanning electron microscope with EDS analysis

By concentrating a high-energy electron beam, the scanning electron microscope produces a range of signals at the surface of solid objects. Zinc oxide nanostructures’ surface appearance and dimensional characteristics were examined using SEM. High-energy electron beams are used to scan samples, and changes in the V-I supply are used to monitor the magnification power (Manjunatha et al., 2019). SEM and EDS tests are essential for characterization and frequently combined, functioning as an imaging tool for nanoparticles and providing details on surface morphology, particle sizes and shapes, imperfections, and other metallographic details. SEM captures the microstructure image of materials and EDS identifies the elemental composition of the material (Al-Ogaili and Almahdawi, 2023).

### 2.7 X-ray diffraction analysis (XRD)

X-ray Diffraction is a highly effective analytical technique that allows for the detection and quantification of crystalline forms of chemicals in powder and solid samples, without any associated risks. XRD uses the strength of the diffraction peaks to analyze a sample’s phases to provide semi-quantitative information about those phases (Relative Phase Abundance and Crystallinity Index). When waves come into contact with a regular structure that has a repetition distance similar to the wavelength, an interesting phenomenon called diffraction takes place (Manjunatha et al., 2019). According to a scientific study, it has been observed that as the temperature at which a sample is calcined increases, the size of the crystals also increases (Hedayati, 2015).

### 2.8 Preliminary qualitative phytochemicals screening

The initial phytochemical analysis was done to confirm that the extract contains the beneficial secondary metabolites. A wide range of phytochemicals were examined, including alkaloids, flavonoids, saponins, steroids, and many more (Mace, 1963; Harborne, 1998; Edeoga et al., 2005; Onwukaeme et al., 2007; De et al., 2010; Gaire et al., 2011; Sunil et al., 2012; Shaikh et al., 2023).

### 2.9 Effect of temperature on the synthesis of nanoparticles

In reaction as the temperature rises, the average size of nanoparticles also rises (Qu et al., 2006). The rise in temperature induces an increase in the size of ZnONPs, indicating that the rate of metal ion reduction increases with temperature. Since high temperature leads to high reaction kinetics (Mohammadi and Ghasemi, 2018).

One of the elements affecting the formation of zinc oxide nanoparticles is temperature. The sample was synthesized at 30°C, 40°C, and 60°C for which UV absorption spectra of the nanoparticles were obtained by which the effect of temperature was studied.

### 2.10 Effect of pH on the synthesis of nanoparticles

ZnO nanoparticles properties are greatly influenced by the pH level used during their manufacture. Due to the low concentration of hydroxyl ions (OH), which impedes the processes of condensation and hydrolysis when the pH is acidic (pH 7), ZnO nanoparticles tend to be smaller. Little effect is seen on the surfaces of nanoparticles at neutral pH (pH = 7). In contrast, an alkaline pH (pH > 7) promotes crystallisation and results in the creation of smaller ZnO nanoparticles because of the high concentration of OH^−^ ions (Khan et al., 2013; Shaban et al., 2022). The pH level employed during the manufacture of ZnO nanoparticles strongly influences their properties. It stresses the regulated, methodical production process needed to modify the pH level and affect the attributes of ZnO nanoparticles. This underscores the importance of precise and reproducible methods in achieving the desired characteristics of the nanoparticles.

To investigate that pH influences synthesis, pH was set at 6,8 and 11 by using 1M HCl and 2M NaOH respectively and then the absorption was measured at 200–800 nm and the effect of pH on nanoparticles was studied.

### 2.11 Biomedical applications

#### 2.11.1 Antimicrobial activity

To measure the efficacy against microbes, the disc diffusion method was employed. Antibacterial testing was carried out employing a bacterial solution spread out over nutrient agar utilizing the disc diffusion method. Whatman’s filter paper was used to prepare the discs, which were then immersed in the test solution for three to 4 hours. After that, the disc was put on the Nutrient agar plate’s surface using sterile forceps, and the plates were incubated for a whole day at the right temperature and the zone of inhibition was assessed. Each organism was inoculated in three plates, and the mean was calculated for the measurement (El-Moneim, 2020).

#### 2.11.2 Antifungal activity

*Candida* strain was used to check the antifungal activity. Fluconazole was used as standard antifungal control (Shaban et al., 2022; Khan et al., 2013; Sukri et al., 2019. Potato Dextrose media was prepared and poured into the agar plate then the inoculated culture was spread over the plate. After preparing the discs using Whatman’s filter paper, they were immersed in the test solution for three to 4 hours. Next, the disc was carefully positioned on the agar plate using sterilized forceps. The plates were then incubated for 24 h at the appropriate temperature. We measured the zone of inhibition to assess the antifungal activity (El-Moneim, 2020).

#### 2.11.3 Antioxidant activity

The DPPH (2,2-diphenyl-1-picrylhydrazyl) technique was used to measure the antioxidant activity of biosynthesized ZnONPs [2]. A hydrogen radical or an electron must be accepted by DPPH, a stable free radical, in order for it to change into a stable diamagnetic molecule. DPPH interacts with an antioxidant that has the potential to give hydrogen and become reduced [35]. The standard utilised to assess antioxidant capability was ascorbic acid (Mishra et al., 2012; Kokila et al., 2016; Vijayakumar et al., 2016; Raut et al., 2021). Different concentrations (50–500 μg/mL) of the extract were added to 1.0 mL of DPPH (0.135 mM) that had been produced. After shaking the reaction mixture and letting it sit at room temperature for 30 minutes, the absorbance at 517 nm was determined (Kokila et al., 2016; Awadh et al., 2022; Muddapur et al., 2022).

#### 2.11.4 Cytotoxic activity against prostate cancer PC3 cells

The current study investigated the impact of ZnONPs on the survival of prostate cancer cells (PC-3) using the MTT colorimetric assay. First, the monolayer cell culture was trypsinized and the cell count was adjusted to 1.0 × 10^5^cells/mL using Dulbecco’s Modified Eagle Medium (DMEM) containing 10% Fetal Bovine Serum (FBS). The cells were then transferred to 96-well microliter plates for growth using Falcon, Becton–Dickinson, Franklin Lakes, NJ, United States. Following a 24-h incubation period, the media was replaced with fresh media containing various concentrations (20, 40, 60, 80, and 100 μg/mL) of the test sample. The assay was concluded after 24 h. The medium was eliminated and replaced with 200 µL of dimethyl sulfoxide (DMSO). The resulting formazan was quantified at 595 nm using a Model 680 Micro plate reader from Bio-Rad Laboratories, Inc., located in Hercules, CA, United States. The percentage growth inhibition was determined by applying a specific formula. The concentration of the test drug required to inhibit cell growth by 50% (IC_50_) was obtained from the dose-response curves for each cell line. This assay relies on the reduction of MTT by the mitochondrial dehydrogenase of intact cells, resulting in the formation of a purple formazan product (Ghasemi et al., 2021).

Inhibition Percentage=OD of Test sample÷OD of control×100

#### 2.11.5 *In vitro* wound healing migration assay

In this study, we examined the effects of a specific concentration of ZnONPs on the spreading and migration properties of L929 cell line cells. At first, the cells were cultured in 12 well animal cell culture plates using DMEM media supplemented with 10% FBS and 2% penstrip antibiotic. After achieving full cellular growth with a density of approximately 50,000 cells, a scratch was created using a sterile 100 μL plastic pipette tip on the monolayer of cells. The removal of cell debris was facilitated by the use of a phosphate-buffered saline (PBS) solution. In addition, the cells underwent treatment with various polymer samples of known concentration. Untreated cells were used as the negative control, while standard ascorbic acid was used as the positive control. The cells were incubated for 24 h at 37°C with 5% CO_2_. The cell layers that were scratched were incubated at various time intervals, including 0 h, 12 h, and 24 h. The cells were photographed and utilized to investigate the relative migration of cells and the closure of wounds. The gap distance was measured using MagVision Software with a calibration at 4X resolution (Pijuan et al., 2019). The formula used to calculate the wound closing and the degree of migration was as follows:

$$\text{Wound closure }\left(\%\right)=A_{0h}\;‐A_{\text{Th}}/\;A_{0h}\times 100\text{Rm}\left(\text{Rate of migration}\right)=W_{i}–W_{f}/T$$.

These included the initial area of the wound (A0h), which was measured immediately after scratching, and the area of the wound (ATh) measured at different time points after the scratch was made. The rate of migration (Rm) was also calculated, representing the speed at which the wound closed in mm per hour. Additionally, the initial (Wi) and final (Wf) widths of the wound were measured, and the duration of migration T) was recorded in hours.

#### 2.11.6 *In vivo* wound healing activity

##### 2.11.6.1 Experimental animals and grouping

Wistar rats of both sexes, weighing approximately 200 g, were utilized in all experiments. The animals were maintained in controlled conditions at a temperature of around 22°F. Their enclosures consisted of polypropylene cages lined with husk, which were replaced every 24 h. The rats had unrestricted access to food and water. The rodents were provided with a standard pellet diet. After obtaining approval from the Najran University Scientific Research Ethical Committee (443–41-49631-DS), the team proceeded with conducting the animal experiments. The three groups of animals were selected randomly with six animals (n = 6) in each group: Group I received no treatment (control-only ointment base); Group II utilized ZnONPs ointment (5% W/W); and Group III utilized standard povidone-iodine ointment (5% W/W).

##### 2.11.6.2 Formulation of ZnONPs loaded ointment

A formulation was created based on a formula found in British pharmacopoeia (Pharmacopoeia, 1988). The ingredients used included white soft paraffin, cetostearyl alcohol, hard paraffin, and wool fat. Method: The ingredients were added in a specific sequence, taking into account their respective melting points. First, 5 g of cetostearyl alcohol was added, followed by 85 g of white soft paraffin, and finally 5 g of wool fat. This resulted in the creation of a 100 g simple ointment base. The components were melted together in a water bath while being stirred constantly until they formed a homogeneous mixture. The mixture was taken off the heat and stirred until it cooled down to room temperature. An ointment was formulated by mixing 5 g of *W. bifurcata* ZnONPs with a portion of the simple ointment base, resulting in a 5% (w/w) ointment. The remaining ointment base was carefully incorporated and thoroughly blended. The ointment was administered externally to the wounds for a continuous period of 21 days during the study.

##### 2.11.6.3 Excision model wound healing activity

Surgical intervention was performed on anesthetized Albino rats, following the established protocol for the excision wound model (Ozay et al., 2018). Rats were given anesthetics to induce anesthesia prior to inflicting the wound injury. The subjects received a dose of 80 mg/kg of ketamine and 5 mg/kg of diazepam intraperitoneally. A circular wound, measuring approximately 500 mm and 2 mm in depth, was carefully made on the shaved portion of the upper back. Following this, ointment containing ZnONPs (test group) and ointment without any medication (control group) were applied topically on a daily basis, as described in the grouping and dosing section. The day the injury began was designated as day 0. At certain time intervals following the creation of the wound, we carefully monitored the healing process, noting the gradual closure of the wound area and the subsequent development of new epithelial tissue. The percentage of the extracts’ wound contraction effect was determined by utilizing an equation, which took into account the initial size of the wound. We determined the length of time it took for the wound to fully heal by monitoring the number of days it took for the raw tissue to disappear after the shedding of dead tissue (Alsareii et al., 2023).

##### 2.11.6.4 Determining the hydroxyproline content

Following a 3-week duration of the experiment, the researchers evaluated the hydroxyproline levels in the excised wound tissues. The tissue samples were dried in a high-temperature oven set at 60°C. Later on, the samples were subjected to hydrolysis for 4 hours at a high temperature of 130°C using a potent hydrochloric acid solution. After adjusting the hydrolysates to a pH of 7.0, an oxidation process was conducted using Chloramine-T for a period of 20 min. The experiment was completed after 5 min by adding 0.4 M perchloric acid, and then using Ehrlich’s reagent to produce color at 60 °C. Following a meticulous agitation process, the samples underwent analysis utilizing an ultraviolet spectrophotometer set at a wavelength of 557 nm. The concentration of hydroxyproline in the tissue samples was determined by using a standard curve of pure L-hydroxyproline (Woessner, 1961).

##### 2.11.6.5 Histopathology

At the end of day-21, the animals were administered Ketamine hydrochloride (50 mg/kg, i. p.) to induce anesthesia before being euthanized. Following the procedure, the samples of the wound tissue and the surrounding healthy tissue were collected. After being fixed in 10% formalin, the collected samples were subjected to a routine histopathological tissue examination. An analysis of the wound tissue specimen was conducted using a staining technique called Hematoxylin-eosin, and observed under a light microscope. An analysis was conducted on sections of the wound tissue sample. These sections were treated with a specialized dye that specifically targets collagen fibers, referred to as Van-Gieson stain. The levels of collagen in the tissue were then assessed using a microscope.

##### 2.11.6.6 Statistical analysis

The data were statistically analyzed using one-way analysis of variance and Tukey’s *post hoc* test; all values are shown as the mean and standard error of the mean (SEM). When *p* < 0.05, statistical differences were deemed significant.

## 3 Results and discussion

### 3.1 Characterization of synthesized ZnONPs

#### 3.1.1 UV-visible spectroscopy

**FIGURE 1:**
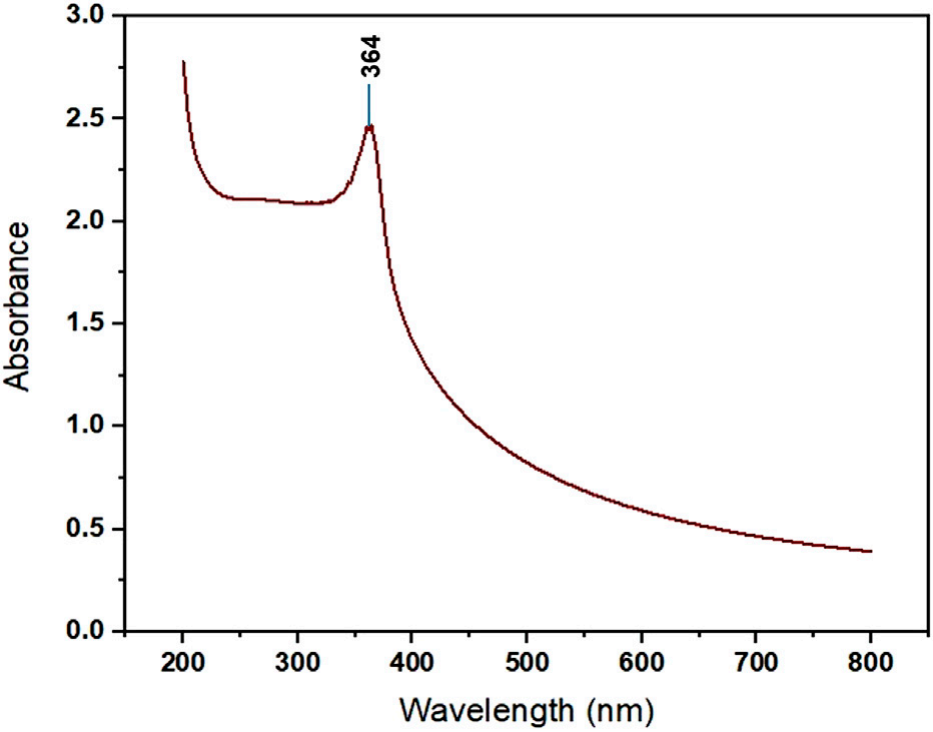
UV Absorption Spectrum of ZnONPs synthesized from *Wodyetia bifurcata* fruit peel extract.

The synthesis of ZnONPs from the fruit peel extract of the Foxtail palm was confirmed by the absorbance band at 364 nm (Figure 1). Vijayakumar et al., reported that ZnO nanoparticles have been studied by UV visible absorption at 300–1000 nm wavelength and reported an absorption peak at 352 nm (Vijayakumar et al., 2016).

#### 3.1.2 FTIR analysis of synthesized zinc oxide nanoparticles

**FIGURE 2:**
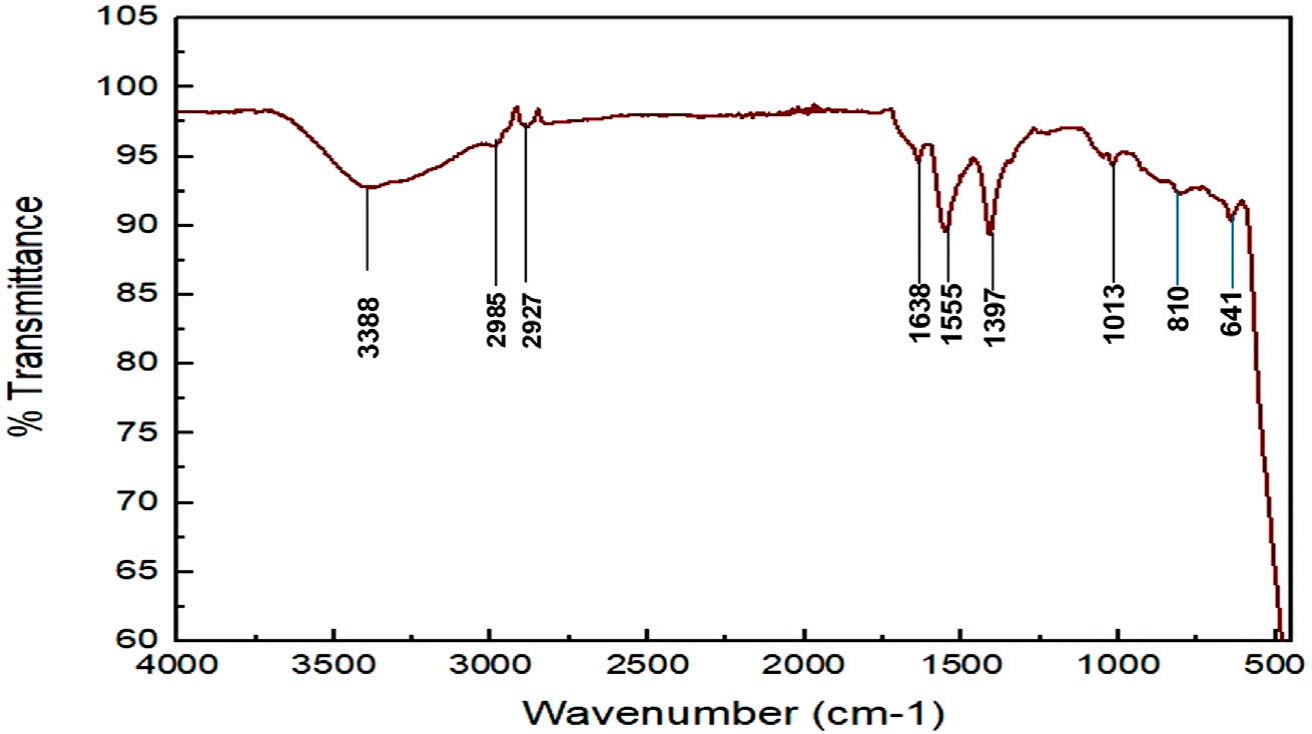
FTIR analysis of ZnONPs synthesized from *Wodyetia bifurcata* fruit peel extract.

The various operating groups involved in the creation of ZnONPs are identified using FTIR. As shown in Figure 2 and Supplementary Table S1 (Supplementary Material), the broad peak at 3,388 cm^-1^ denoted the presence of -OH stretching vibrations. The strong absorption bands at 2,985 and 2,927 cm^-1^ can be ascribed to the stretching mode of C-H bonds of *W. bifurcata* Fruit Peel Extract. The strong band at 1,638 is attributed to the C=C stretch in aromatic ring and C=O stretch in polyphenols. The band observed at 1,502–1,565 cm^-1^ corresponds to the carbonyl group of flavonoids. Moderate levels of absorption in the region covering 1,469–1,384 cm-^1^ imply the presence of an aromatic ring. The peak at 1,397 results from aromatic amine. The peak at 1,013 is due to Monosubstituted Aromatic Ring. FTIR spectrum also shows the characteristic vibration band at 810, and 641 cm^-1^, which was correspond to E2 mode of hexagonal ZnO wurtzite structure. The FTIR spectrum of zinc oxide nanoparticles absorb at 441–665 cm^−1^ (Singh et al.)

#### 3.1.3 Scanning electron microscope (SEM) imaging

**FIGURE 3:**
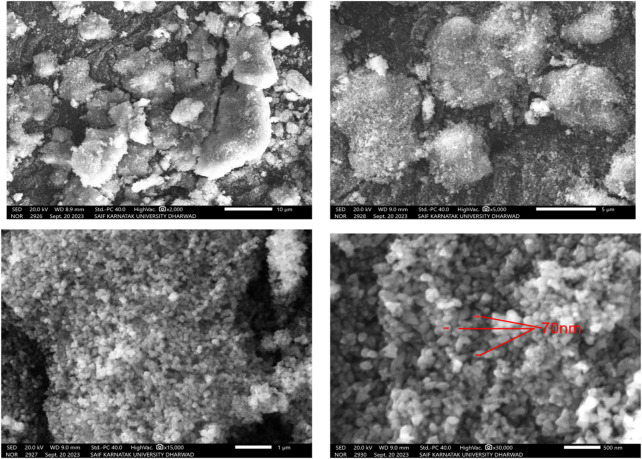
SEM images of ZnONPs synthesized from *Wodyetia bifurcata* fruit peel extract.

SEM is a powerful technique commonly used for the characterization of nanoparticles, including ZnONPs. The form and size of the synthesised ZnO nanoparticles (ZnONPs) in this study are revealed by the SEM examination, and these findings are in close agreement with previous research in the field. The ZnONPs have a mostly spherical form and a discernible propensity to aggregate (Figure 3). The detected particle sizes are approximately 70 nm, a feature that is remarkably consistent with multiple investigations that have been published before. A detailed investigation of the synthesis and characterisation of ZnONPs was carried out by Raut et al., in 2021. According to their findings, the nanoparticles had a significant propensity to aggregate and a spherical morphology. In their investigation, the particle sizes varied from 20 to 50 nm. Comparable to the clustering seen in the current work, the agglomeration exhibited in their SEM pictures highlights a shared characteristic among ZnONPs synthesised using similar methods (Raut et al., 2021).

#### 3.1.4 SEM with energy dispersive X-Ray spectroscopy

**FIGURE 4:**
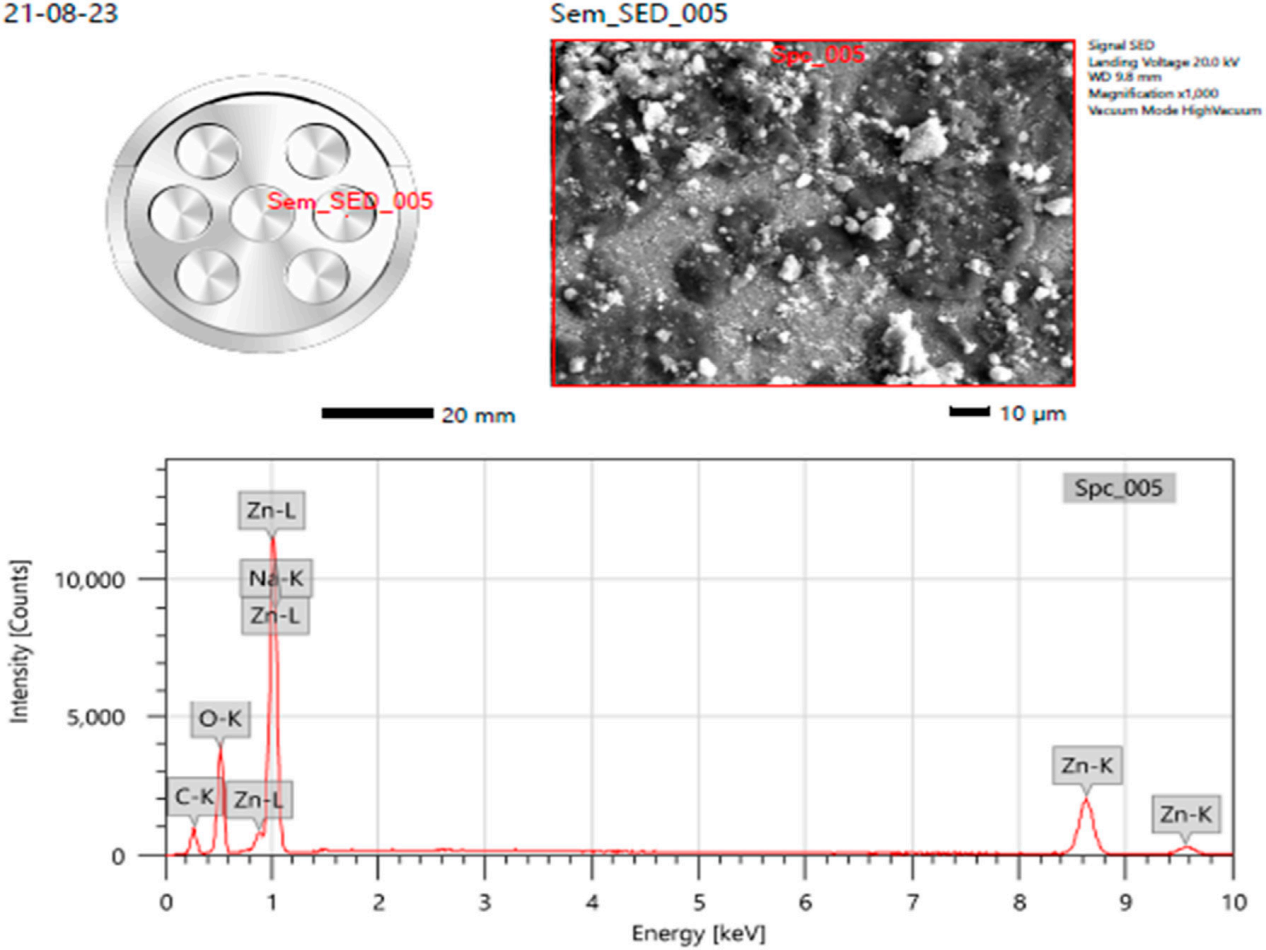
Elemental analysis (EDS) of ZnONPs synthesized from *Wodyetia bifurcata* fruit peel extract.

With the purpose of providing additional insight into the topographies of ZnO NPs, energy dispersive X-ray (EDX) analysis was used to explore the sample. The samples’ EDX spectra from the SEM analysis show that the sample made using the previously described approach contains a clean ZnO phase (Vidya C. et al., 2013). The EDS spectra peaks for zinc (Zn), oxygen O), and carbon C) in the below image (Figure 4) verified the generation of ZnONPs, and the EDS revealed that 17.81% weight of the synthesized ZnONPs are present (Figure 4). Vijayakumar et al., 2016, reported that the groups found in the heterocyclic rings of the biomolecules present alongside the ZnO nanoparticles are responsible for the existence of additional elements, as shown by the EDX analysis. The atomic percent of zinc was 48% accompanying with the 25% for oxygen atomic percent which gives the 2:1 ratio for Zn and O, correspondingly.

#### 3.1.5 XRD analysis

**FIGURE 5:**
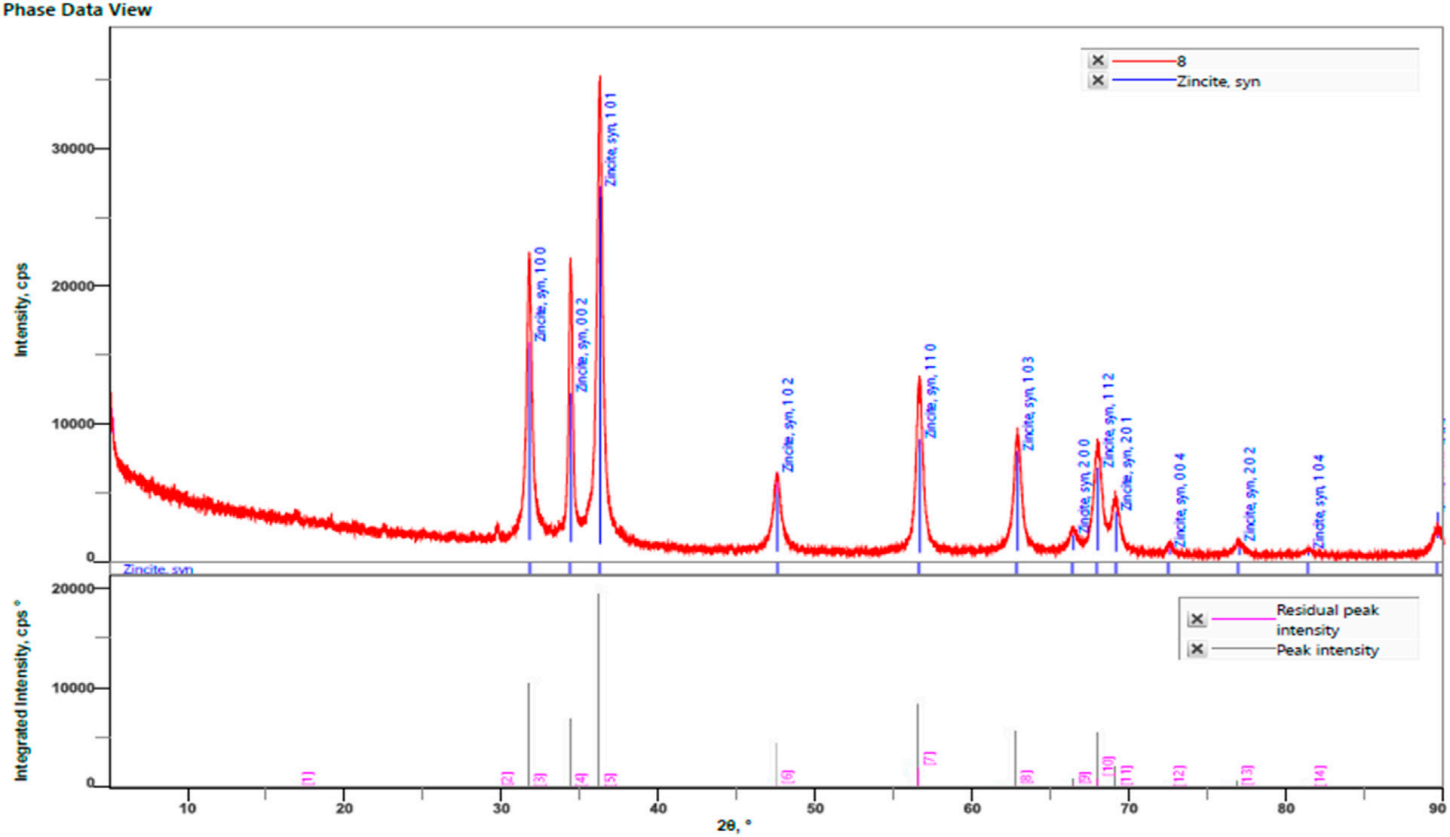
XRD analysis of ZnONPs synthesized from *Wodyetia bifurcata* fruit peel extract. (Card number- JCPDS-36-1451).

The primary applications of X-ray diffraction (XRD), a potent analytical method, are phase identification, crystalline structure identification, and the determination of certain structural parameters like strain, lattice constants, and crystallite size. The phases, structures, and crystal orientations of the nanoparticles at 31.74, 34.38, 36.22, 47.52, 56.55, 62.74, 66.45, 67.98, 69.11, 72.49, 76.84, and 81.46° are examined using XRD (Figure 5). Diffraction was seen and the lattice planes (100), (002), (101), (102), (110), (103), (200), (112), (201), (004), (202), (104). Niranjan Bala et al. (2015) reported that ZnO production began at 30°C and that sample crystallinity increased as the temperature increased (Bala et al., 2015). The XRD pattern provided corresponds to synthetic zincite (ZnO), as indicated by the matching peaks and their respective Miller indices. The pattern shows a good fit between the experimental data and the reference pattern, with low residual peak intensities (JCPDS-36–1,451). This confirms the crystalline nature and phase purity of the ZnO sample analysed.

The Debye -Scherrer equation was utilized to predict the crystal size of the ZnO-NPs. Using Debye–Scherrer’s formula, the particle size of the ZnO-NPs was determined to be 26.58109 nm (Supplementary Table S2).

### 3.2 Preliminary phytochemicals screening

Supplementary Table S3, shows the phytochemical analysis of fruit peel extract. The extract showed the presence of flavonoids, saponins, steroids, triterpenoids, and resins. Recent studies shown that plant extract including phenols and flavonoids is utilized to create ecologically safe metal oxide nanoparticles (Awadh et al., 2022; Muddapur et al., 2022)]. According to earlier research, these ingredients serve as stabilising and reducing agents in the environmentally friendly production of zinc oxide nanoparticles. The zinc compounds can be reduced and stabilised into ZnONPs by the OH groups found in phenols and flavonoids.

### 3.3 Optimization

#### 3.3.1 Effect of temperature and UV-Vis analysis

**FIGURE 6:**
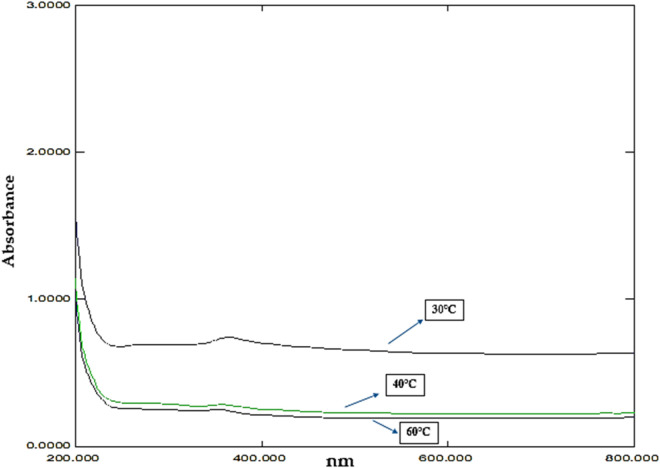
UV absorption graph showing the effect of temperature.

Distinct peaks at 364nm, 358 nm, and 354 nm were obtained in the UV spectroscopy data for the ZnO-NPs synthesized at 30°C, 40°C, and 60°C, respectively (Figure 6; Supplementary Table S4). Muddapur et al., reported that the size of the nanoparticles decreases because of the increase in temperature, which also increases the generation of mono-dispersed, smaller-sized nanoparticles by increasing the rate of reaction and the molecules’ activation energy (Raut et al., 2021). The UV-Vis absorption spectra of ZnO nanoparticles synthesized at varying temperatures (30°C, 40°C, and 60°C) reveal a decrease in absorbance and a sharpening of the absorption peak with increasing temperature. This observation suggests a reduction in nanoparticle size as the temperature rises, which is characterized by narrower and more defined Surface Plasmon Resonance (SPR) waves. This finding appears to contradict the methodology section, which suggests that ZnONPs increase in size with higher temperatures. However, several studies support the observed trend, indicating that higher synthesis temperatures can lead to smaller nanoparticles due to enhanced nucleation rates. Ristić et al. (2005) reported that increased temperatures favour nucleation overgrowth, resulting in smaller ZnO nanoparticles. Furthermore, Wahab et al. (2014) demonstrated that careful control of synthesis temperature significantly impacts the size and uniformity of ZnONPs.

#### 3.3.2 Effect of pH

**FIGURE 7:**
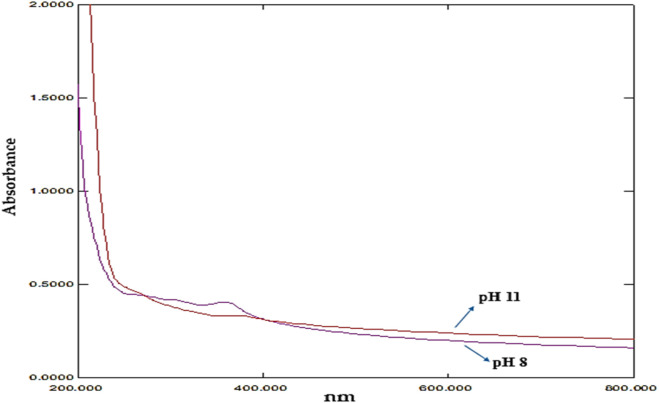
UV absorption graph showing the effect of pH.

At pH six the peak was not observed and at 8 and 11 the peak was observed at 358nm and 264 nm Figure 7 and Supplementary Table S5). Awadh et al., found that the size of ZnO NPs generated by diverse approaches is influenced by pH (Awadh et al., 2022). The size and morphological alterations of ZnO-NPs are responsible for the variation in the UV-Vis absorption peaks with pH. Higher hydrogen ion concentrations at lower pH levels can cause aggregation and inadequate deprotonation during synthesis, which can result in the creation of bigger, less uniform nanoparticles. As may be seen at pH 6, this frequently leads to larger, less distinct absorption peaks or perhaps the absence of any peaks at all. Higher pH levels, on the other hand, promote more thorough deprotonation due to the reduced hydrogen ion concentration, which results in the creation of smaller, more homogeneous nanoparticles with clearly defined absorbance peaks. The peaks for pH eight and pH 11 at 358 nm and 264 nm, respectively, clearly show this Singh et al. (2024). An additional explanation for the sharper peaks seen at higher pH levels is the nanoparticles’ enhanced crystallinity and narrowed size distribution. Sharpness in the absorption spectra is a result of more uniform particle sizes produced by higher nucleation rates when pH rises. The research conducted by Sanju Singh et al. (2024) supports this occurrence.

### 3.4 Biomedical applications

#### 3.4.1 Antimicrobial activity

**FIGURE 8:**
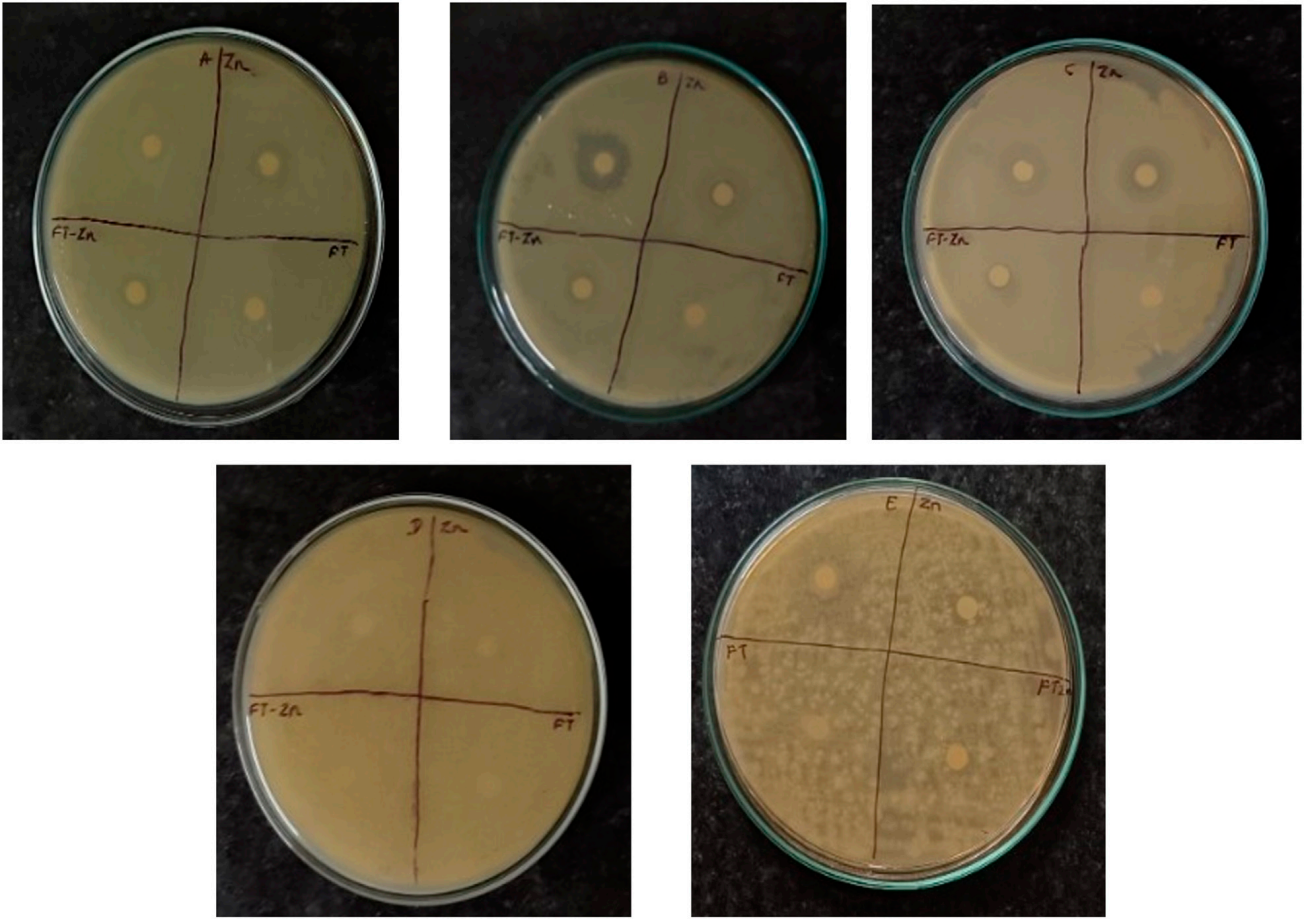
Antibacterial activity. A- *Escherichia coli;* B- *Bacillus Subtilis* C*- Pseudomonas aeruginosa;* D*- Zymomonas mobilis;* E*- Staphylococcus aureus;* Zn-Zinc Nanoparticals; FT-Foxtail palm fruit, FT-Zn -Synthesized ZnONPs + Extract.

The disk diffusion method was used to evaluate antimicrobial activity. The results showed the effect of nanoparticles (1 mg/mL) on Gram-positive and Gram-negative bacteria. Synthesised ZnO-NPs showed a zone of inhibition for *Escherichia coli is* typical Gram-negative bacterium that is frequently employed as a model organism in microbiology. It is an important target for antibiotic testing since it frequently causes UTIs and foodborne diseases. *, Bacillus subtilis* is a soil-dwelling bacterium that is well-known for its spore-forming capacity. Because of its robustness and simplicity of growing, it is frequently utilized in laboratory experiments. In research on antibiotics, it acts as a model for Gram-positive bacteria.*, Pseudomonas aeruginosa* is a Gram-negative bacterium linked to a number of diseases, particularly in people with weakened immune systems. Because of its well-known antibiotic resistance, this bacterium is essential for evaluating novel antimicrobial drugs.*,* Gram-negative *Z. mobilis (Zymomonas mobilis)* is a bacterium of importance because it plays a part in the synthesis of bioethanol. Current study focuses on its metabolic routes, resistance mechanisms, and antimicrobial agent response. and a zone of inhibition was not observed for Gram-positive *S. aureus, or Staphylococcus aureus,* is a bacterium that can cause a variety of infections, from simple skin infections to serious illnesses including sepsis and pneumonia. In investigations on antibiotic resistance, it is a crucial pathogen. (Figure 8; Table 1). Vijayakumar et al., reported that Gram-positive bacteria show more convergent ZnO nanoparticle penetration into cell membranes than their negative counterparts because of the presence of peptidoglycan layers (Vijayakumar et al., 2016) [41]. When exposed to light, ZnO nanoparticles can produce reactive oxygen species (ROS) such as superoxide anions, hydroxyl radicals, and hydrogen peroxide. These ROS have the ability to induce oxidative stress, which damages the proteins, lipids, and DNA found in bacterial cells (Padmavathy et al., 2008).

**TABLE 1:** Antibacterial activity against pathogenic bacteria.

Bacterial strains	Zone of inhibition in millimetre (mm)			
	Standard (*Ampicillin*)	ZnONPs	(FT)Extract	FT + ZnONPs
*E. coli (*ATCC 25922)	8 ± 0	10 ± 0.272	8 ± 0.118	7
*B. Subtilis* (ATCC 122264)	19 ± 0.471	7 ± 0.36	-	8 ± 0.118
*P. aeruginosa (*ATCC 27853)	13 ± 0	14 ± 0.72	-	7 ± 0.136
*Z. mobilis (*ATCC 31821)	-	7 ± 0.272	11 ± 0	10 ± 0.136
*S. aureus (*ATCC 25923)	21 ± 0	11 ± 0.272	-	-

The experiment was performed in triplicate and the values are expressed as Mean ± SEM.

#### 3.4.2 Antifungal activity

**FIGURE 9:**
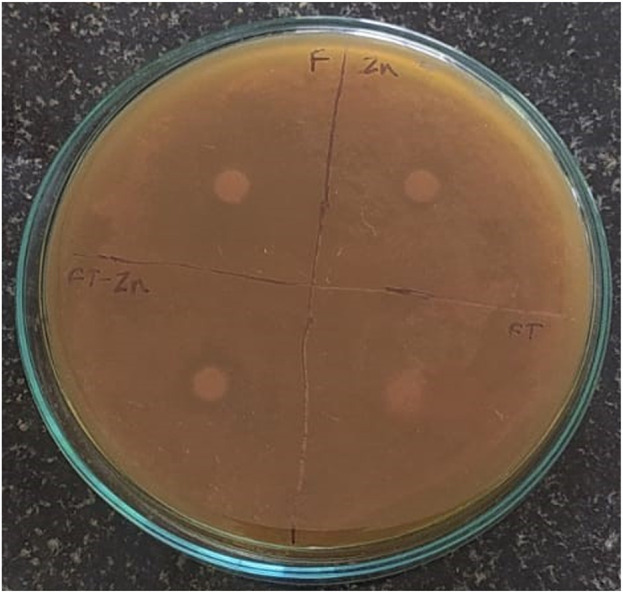
Antifungal activity observed against *Candida albicans.* Zn-Zinc Nanoparticles, FT-Foxtail palm fruit, FT-Zn–Extract + ZnONPs.

The antifungal activity was evaluated using the Disk Diffusion method. A fungal strain, *Candida albicans*, was used in the study. A zone of inhibition measuring 12 mm was observed for the plant extract + ZnONPs, while the ZnONPs showed a ZOI of 9 ± 0.136 mm, and the standard antifungal drug fluconazole exhibited a ZOI of 27 ± 0.27 mm as shown in Figure 9 and Supplementary Table S6.

### 3.5 Antioxidant activity

**FIGURE 10:**
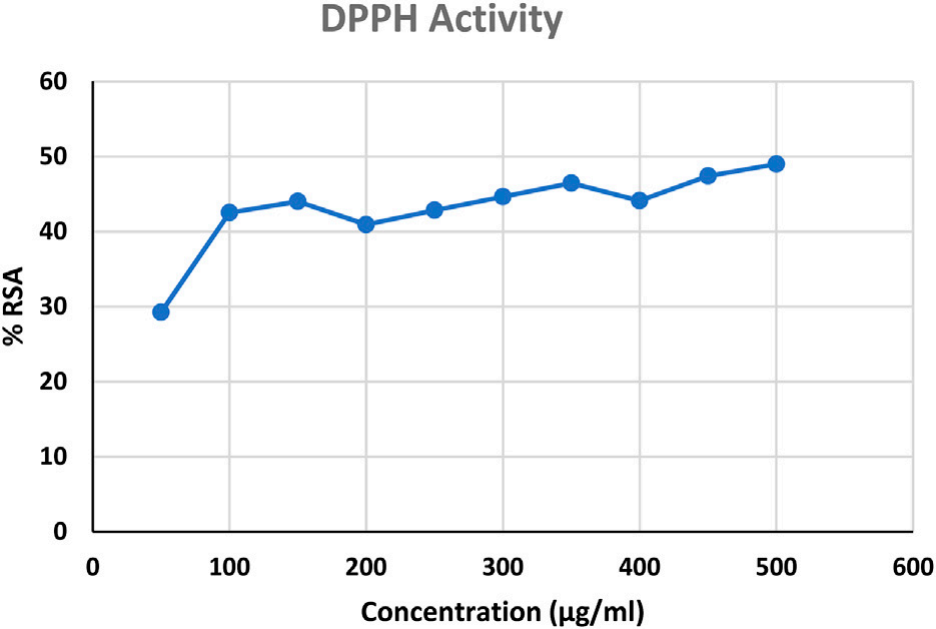
DPPH scavenging activity; RSA-Radical Scavenging Activity.

DPPH scavenging activity was done using ascorbic acid as standard and a bar graph was plotted (Figure 10). The percentage of radical scavenging activity (RSA) of standard ascorbic acid is more than that of the sample. Sharma and Bhat, 2009, studied DPPH absorbance profiles in ethanol, buffered methanol, and methanol with ascorbic acid. Standard antioxidants, such as BHT and propyl gallate, were utilized to compare antioxidant capability and scavenge DPPH radicals (Sharma and Bhat, 2009; Ghasemi et al., 2021).

### 3.6 Cytotoxic activity against PC3 cell line

It is widely recognized that cancer is a significant contributor to mortality rates in humans. Prostate cancer is a commonly diagnosed cancer and a significant cause of cancer-related deaths in men. Its development may be linked to abnormalities in the process of cell death (Rawla, 2019). Therefore, the individuals capable of influencing apoptosis in PC-3 cells could potentially have significant therapeutic implications for prostate cancer. Therefore, it is crucial to create treatments for the management of prostate cancer.

Chemotherapy plays a crucial role in the clinical management of cancer, alongside radiation therapy and surgical procedures. Medicinal plants play a significant role in providing chemotherapy drugs in both traditional and modern systems of medicine worldwide (Desai et al., 2008). Their phytochemicals or extracts have shown promising results in combating cancer, outperforming traditional chemotherapy and hormonal treatments. In recent years, there has been significant interest in the phytochemicals, such as flavonoids, alkaloids, and terpenes, because of their diverse pharmacological properties. These properties include their potential to prevent cancer and their cytotoxic effects (Kumar et al., 2014).

There are various management options to consider for early-stage prostate cancer, such as watchful waiting, surgery, cryosurgery, radiation therapy, and hormonal therapy. Current treatment options, such as radiation therapy and chemotherapy, have limitations due to their impact on healthy cells and the challenge of drug resistance.

The most effective initial treatment for advanced prostate cancers is the surgical or medical ablation of androgens. In the majority of patients undergoing androgen deprivation therapy, the disease will continue to progress until it reaches a stage known as castration-resistant prostate cancer (CRPC). The transition to a hormonal refractory state is a multifaceted phenomenon, encompassing the emergence and expansion of androgen-independent cell clones, as well as the activation of genes that facilitate the survival and proliferation of cancer cells following androgen deprivation (Neal et al., 2007).

The field of cancer nanotechnology holds immense promise in the areas of cancer diagnosis, targeted treatment, and monitoring. Scientists are currently investigating the potential of nanoparticles (NPs) of varying sizes to detect, image, monitor, and treat different types of cancers. One area of great potential in the field of nanotechnology is the development of magnetic nanoparticles (MgNPs). These nanomaterials are biocompatible, super-paramagnetic, chemically stable, and have low toxicity, making them highly promising (Alrushaid et al., 2023).

**FIGURE 11:**
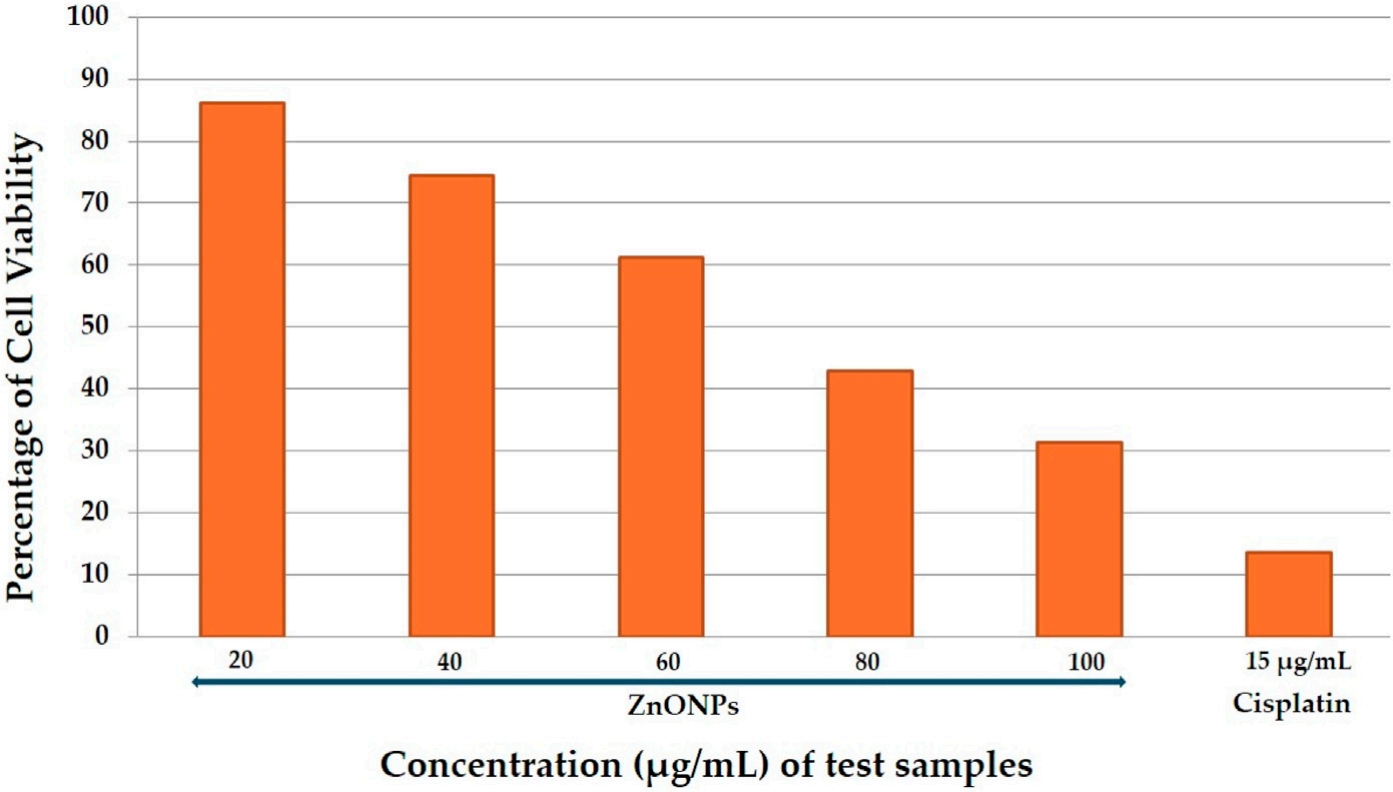
Percentage of cell viability of PC-3 Prostate cancer cells treated by test sample.

**FIGURE 12:**
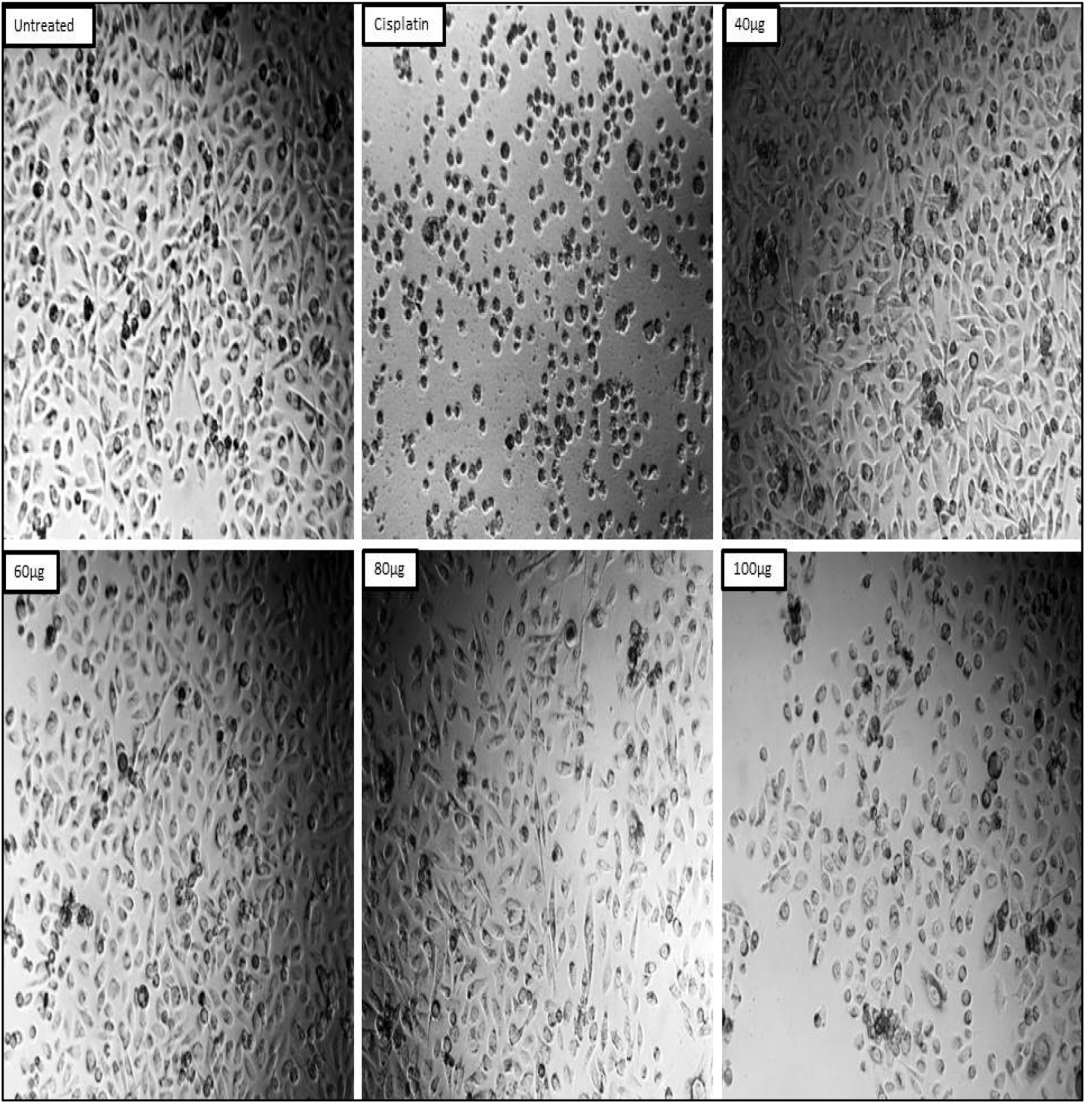
Effects of test sample on the Prostate cancer PC-3 cells.

In the present study the anticancer activity of test sample was tested against prostate cancer PC-3 cell line by MTT cell viability assay along with standard drug cisplatin and untreated group of cells as positive and negative control respectively. The results revealed that the anticancer activity of test sample was seen in dose dependent manner, i.e., with increasing in the concentration the percentage of cell viability was decreased. At initial concentration 20 μg/mL the percentage of cell viability was observed to be 86.14% and at its higher concentration (100 μg/mL), the percentage of cell viability was decreased to 31.98% (Table 2; Figure 11). IC_50_ concentration from the calibration graph was found to be 73.05 µg. In case of standard drug cisplatin, the percentage of cell viability was observed to be 13.59 ± 0.004. Microscopic study revealed that in treated group of cells there is observation of detachment of cells, cell turgidity, and cell shrinkage and cell elongation. As the concentration of test sample was increased there were morphological changes in the cells (Figure 12).

**TABLE 2:** Percentage of cell viability of PC-3 prostate cancer cells treated by ZnONPs.

S. No	Treatments	Concentrations in µg/mL	Percentage of cell viability
1	Sample-ZnONPs	20	86.14 ± 0.007
		40	74.46 ± 0.002
		60	61.11 ± 0.002
		80	42.94 ± 0.004
		100	31.98 ± 0.002
2	Standard-Cisplatin	15	13.59 ± 0.004

The results are expressed as mean ± standard deviation.

### 3.7 Invitro wound healing activity employing scratch assay

Effective healing of skin wounds is a complex task in the medical field, and there is a growing demand for innovative materials to address this challenge. An injury typically occurs when the skin’s normal function and structure are disrupted (Lazarus et al., 1994).

The most frequent factors contributing to delayed healing or non-healed wounds are wound infection, the presence of bacterial proteins, ischemia, and chronic trauma. It is crucial to select the appropriate materials for wound dressing in order to promote wound healing and minimize the risk of infections. Wound healing is a fascinating biological process that can be divided into four distinct phases. The first phase, known as hemostasis, focuses on closing the wound by promoting blood clotting. This is followed by the inflammatory phase, where proteins and growth factors are secreted to facilitate tissue repair. During the proliferative phase, the focus is on maximizing the healing of the skin, while in the remodelling phase, the emphasis is on the production of collagen, which plays a vital role in providing the necessary strength to the wound (Boateng et al., 2008). Various nanotechnology-based systems have been devised to expedite the process of wound healing by stimulating various stages of the healing process. Furthermore, they have the potential to transport various substances, including natural products, antibacterial and anti-inflammatory agents, as well as growth factors, directly to the areas that require healing (Shi et al., 2010).

**FIGURE 13:**
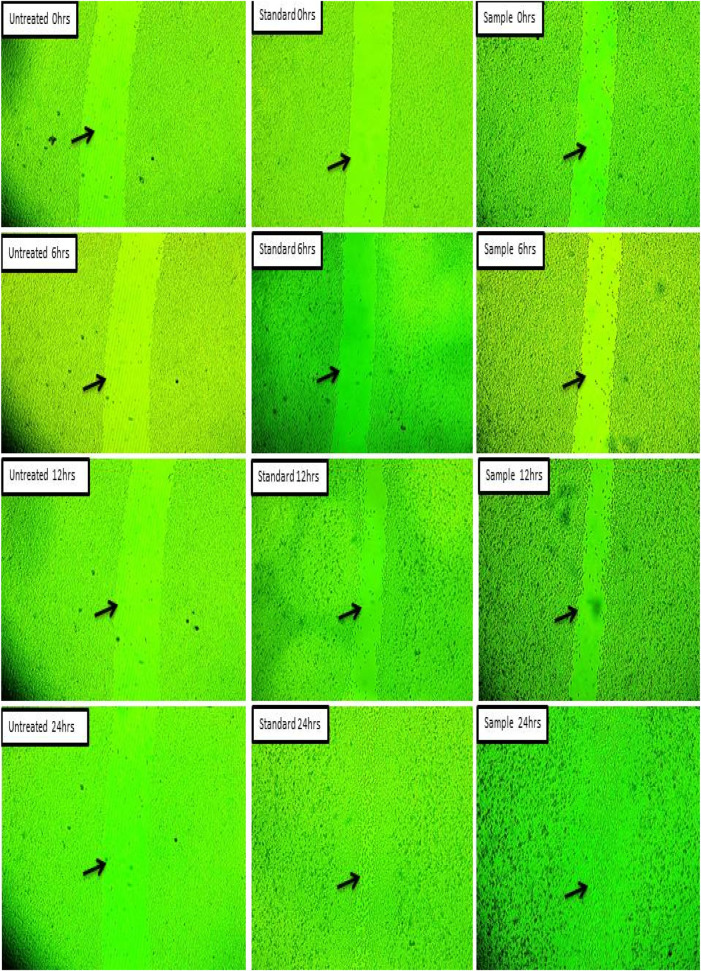
Wound closure study of silver nanoparticles on L929 cell line.

In the present study the test sample was screened for the wound healing activity using L929 cell line along with standard ascorbic acid as a positive control group. The known concentration (15 µg) of test sample and standard ascorbic acid (5 µg) was treated on L929 cell line using standard scratch assay. The results revealed that both cell migration and wound closure have shown appreciable results in both test sample and standard ascorbic acid. In case of standard ascorbic acid the wound closure found to be 93.45%, test sample seen to be 90.81% and for untreated it was seen to be 8.12% (Table 3; Figure 13).

**TABLE 3:** Cell migration of different test samples at different time intervals and percentage of Wound Closure (24 h).

Sl. No	Test sample	Duration (h)	Cell migration (mm)	% wound closure (24 h)
1	Untreated (Control)	6	3.56	8.12
		12	2.31	
		24	1.87	
2	Ascorbic acid (Standard)	6	10.54	93.45
		12	7.32	
		24	3.44	
3	Test sample (ZnONPs)	6	7.82	90.81
		12	5.50	
		24	4.15	

### 3.8 Effect of *Wodyetia bifurcata* ZnONPs on *in vivo* wound healing

**FIGURE 14:**
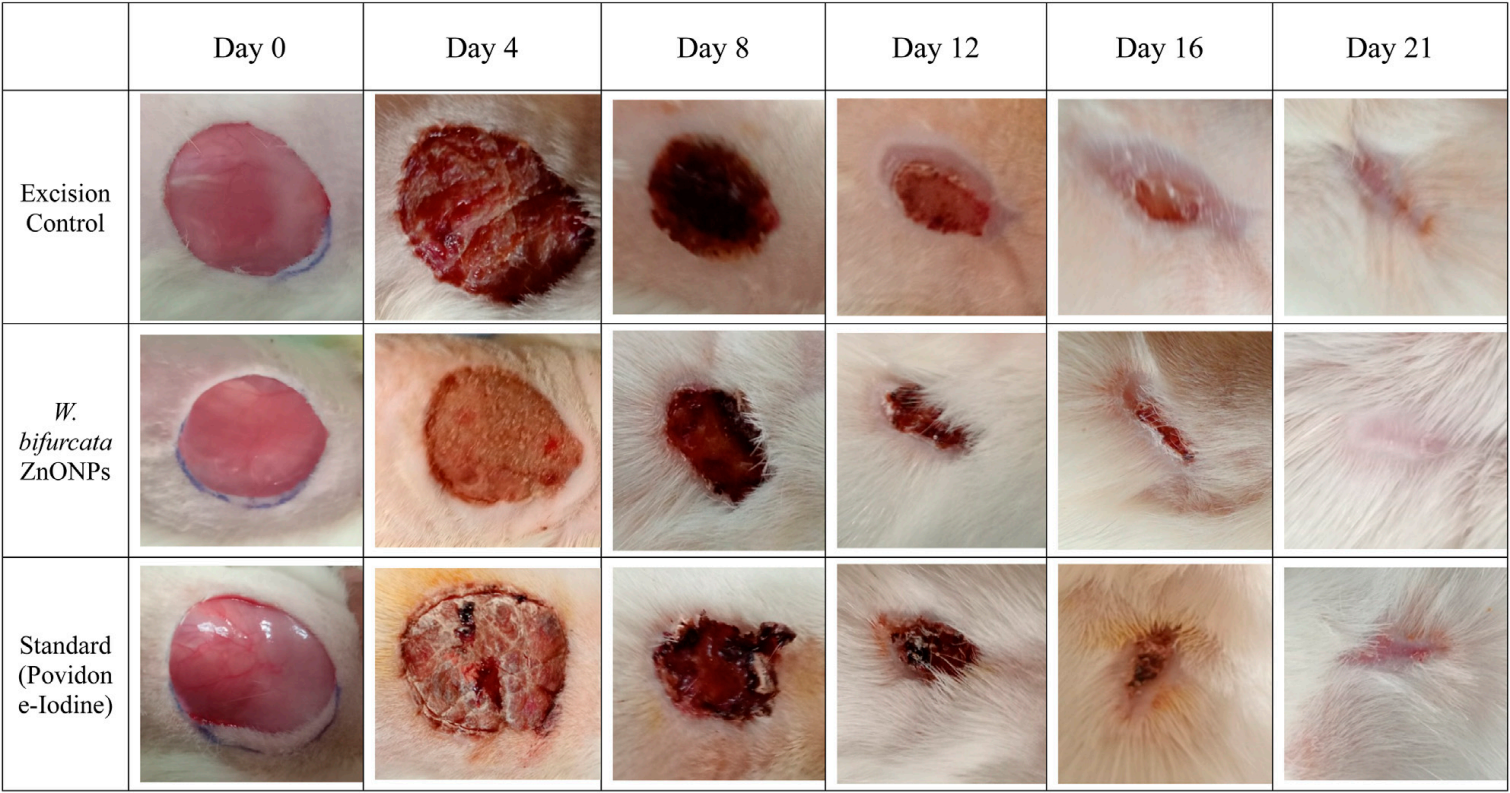
Images showing the impact of *Wodyetia bifurcata* ZnONPs on excision wound model at different time points.

The percentage of wound contraction for *W. bifurcata* ZnONPs and Povidone-Iodine can be found in Table 4. On day 12, a significant (*p* < 0.01) wound contraction was observed in animals that received treatment with ZnONPs compared to the control animals. While, the animals treated with standard Povidone-Iodine ointment showed a significant wound contraction starting from Day 4, with a statistical significance (*p* < 0.01). On day-21, the animals exhibited significant healing of the wound after being treated with ZnONPs and Povidone-Iodine. Animals in the control group took 17.1 days for epithelization, whereas the animals treated with ZnONPs and Povidone-Iodine had much shorter periods of 11.1 and 8.2 days, respectively. The groups treated with ZnONPs and Povidone-Iodine demonstrated a significantly faster rate of complete epithelization compared to the control group, which did not receive any treatment (*p* < 0.01). The wound healing effect of ZnONPs ointment was comparable to that of the Povidone-Iodine ointment (Figure 14). The *in vivo* experiments on wound healing provided additional evidence of the positive impact of ZnONPs. When ZnONPs were applied topically, they showed an impressive ability to enhance wound closure and stimulate tissue regeneration. The nanoparticles have been found to significantly improve the healing process of wounds. This is thought to be due to the promotion of blood vessel genesis, increased production of collagen, and faster regeneration of the outer skin layer. As a result, wound healing is expedited and becomes more efficient.

**TABLE 4:** Effect of *Wodyetia bifurcata* ZnONPs on wound diameter, wound area and percentage wound contraction in an excision wound model.

Groups		Wound diameter (mm)					Area (mm sq)						Percentage wound contraction (%)					
	D0	D4	D8	D12	D16	D21	D0	D4	D8	D12	D16	D21	D0	D4	D8	D12	D16	D21
Excision (Control)	25.5± 0.5	22.5± 0.5	19.5 ± 1.1	15.25± 0.9	11.75± 0.9	8.5± 0.5	510.6 ± 23.1	397.6± 20.3	299.4± 39.5	183.1 ± 22.6	108.9 ± 17.9	56.9 ± 7.7	0	22.1± 0.4	41.2± 7.9	64.04± 5.07	78.7± 2.6	88.8± 1.01
*W. bifurcata* ZnONPs	25± 0.8	21.25± 0.5	16.5 ± 1.118	12.5± 1.2	8± 0.8	3± 0.8	491.01± 32.05	354.6 ±16.8	214.6 ± 33.4	123.6 ± 25.3	50.6 ± 10.2	7.4 ± 3.8	0	27.5± 5.6	55.9± 8.6	74.4± 6.8*	89.6± 2.1***	98.4± 0.8***
STD (Povidone-Iodine)	26± 0.5	19.75± 1.2	15 ± 1.581	11 ± 0.8	2.5± 0.5	0.25± 0.5	510.6± 23.1	307.1± 38.3	178.5 ± 43.01	95.3 ± 14.1	5.1 ± 2.2	0.1 ± 0.3	0	39.7 ± 8.2**	64.7± 9.9*	81.3± 2.2**	98.9± 0.4***	99.9± 0.07***

Values are expressed as Mean ± SEM, for six animals per group. *P< 0.05; **P < 0.01; **P < 0.001 compared with control (ANOVA, followed by *post hoc* tests for multiple comparisons).

#### 3.8.1 Estimation of hydroxyproline content in the healed tissue

**FIGURE 15:**
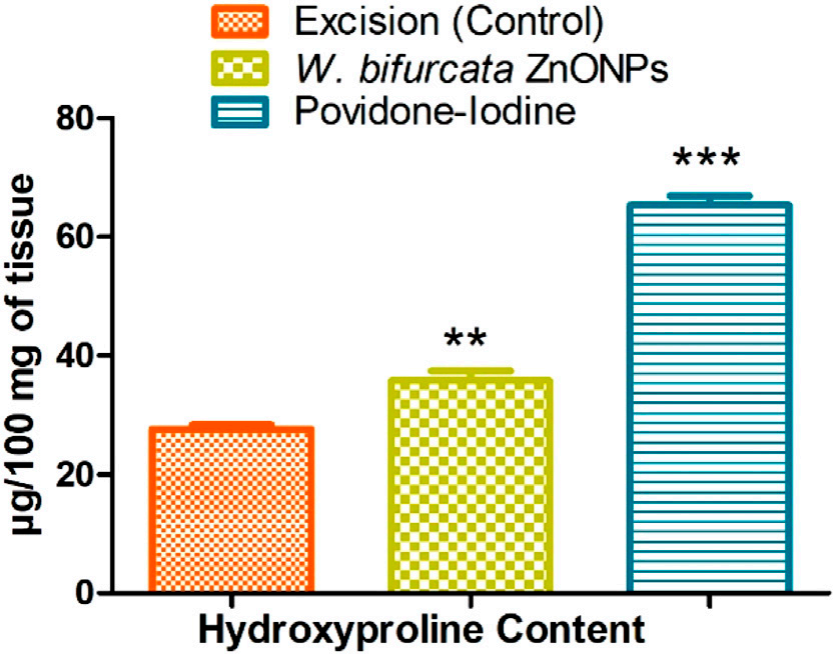
Effect of ZnONPs on Hydroxyproline Content (µg/100 mg of tissue). Values are expressed as Mean ± SEM for six animals per group.**P < 0.01; ***P < 0.001 compared with control group.

Figure 15 demonstrates the effect of *W. bifurcata* ZnONPs on the Hydroxyproline content in the rejuvenated tissue. The hydroxyproline content (µg/100 mg of tissue) in the excision control animals was determined to be 27.62 ± 0.7. The hydroxyproline content in animals treated with ZnONPs was observed to be significantly higher (35.74 ± 1.7) when compared to animals in the excision control group, with statistical significance at *p* < 0.01. Furthermore, the rats that were administered Povidone-Iodine showed a significant rise in hydroxyproline content (65.34 ± 1.5) when compared to the excision control animals. This difference was statistically significant (*p* < 0.001).

Hydroxyproline makes up approximately 13.5% of mammalian collagen. Hydroxyproline and proline play important roles in collagen stability. They allow for severe bending of the collagen helix. A healed tissue produces collagen, a component of developing cells. The concentration of hydroxyproline measures the concentration of collagen. A higher hydroxyproline concentration signifies a faster healing rate for the wound (Dwivedi et al., 2017; Alsareii et al., 2023). Based on the biochemical analysis, the skin samples in the current study showed higher hydroxyproline contents, indicating a potential increase in collagen synthesis and cellular proliferation. Collagen plays a crucial role in providing strength and stability to the tissue matrix, ensuring proper homeostasis and facilitating epithelialization during the restorative process. Therefore, the increased production of hydroxyproline strengthens the healing process and the regenerated tissue (Woessner, 1961).

#### 3.8.2 Histopathology study

**FIGURE 16:**
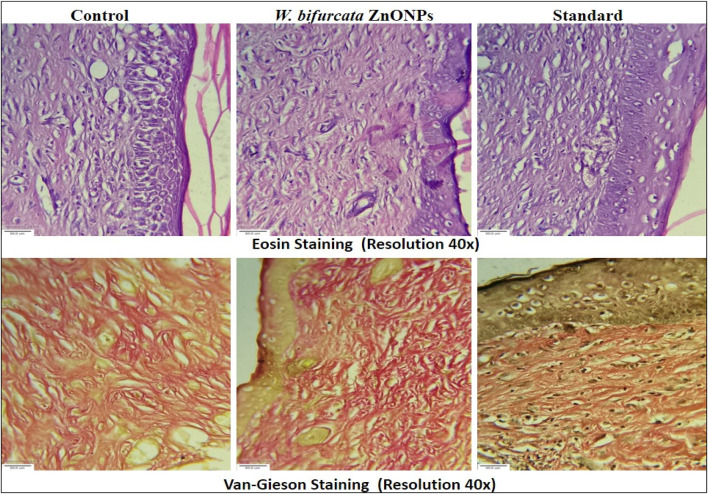
Histopathology images of newly regenerated tissue at day 21 (magnification ×40).

The histopathology study revealed that the collagen fibers were abundant and well-organized in the rats treated with ZnONPs and the standard treatment povidone-iodine ointment (Figure 16). In contrast, notable damage was observed in the untreated group, including dermal edema, congestion, inflammatory infiltration, substantial fibrosis, cellular infiltration, inflammation, and epithelial deterioration. The animals that received treatment with ZnONPs and povidone-iodine exhibited various positive effects on their skin, including the development of a thick epidermal layer, dermal granulation tissue, papillary dermis, sebaceous glands, dermal collagen, neutrophilic infiltration, macrophages, dermal fibroblasts, and hair follicles. In contrast, the untreated control animals did not show these same improvements. Animals that received ZnONPs treatment exhibited no signs of inflammation in the regenerated tissue, identical to the ones treated with the standard drug. The ointment formulation with ZnONPs enhances the development of intricate and well-structured cell formations, which is beneficial for the growth of regenerated skin tissue. Granulation tissue plays a crucial role in supporting the growth of new tissue and promoting the healing process of wounds. Fibroblasts demonstrate a remarkable ability to adapt their shape, which is crucial for carrying out their diverse functions. Throughout the process of wound healing, fibroblasts have the remarkable ability to undergo a transformation into myofibroblasts. This conversion plays a crucial role in their mechanism for fibrosis and their ability to contract and close wounds. Likewise, collagen plays a vital role in the process of skin regeneration. It facilitates the growth of new skin cells and enhances the healing process by promoting cell migration and epithelization from the wound edge. The animals treated with ZnONPs showed an increase in dermal granulation tissue and collagen.

## 4 Conclusion

The current study showed that *W. bifurcata* ZnONPs boosted both *in vitro* and *in vivo* wound-healing potential. The evidence demonstrated that applying *W. bifurcata* to wounds caused significant wound constriction and sped up the healing process. Moreover, the extract was found to have no cytotoxic effects and exhibited strong antimicrobial activity. Regarding the anticancer activity, the ZnONPs demonstrated dose-dependent effects against prostate cancer PC-3 cell line. Overall, the research provides evidence of the potential antimicrobial, antioxidant, anticancer and wound healing properties of the ZnONPs, suggesting its possible application in cancer treatment and wound management. Further studies are warranted to explore the underlying mechanisms and evaluate the safety and efficacy of the test sample in clinical settings.

## Data Availability

The original contributions presented in the study are included in the article/Supplementary Material, further inquiries can be directed to the corresponding authors.
