# Supplementary material for: Green synthesis of zinc oxide nanoparticles from Wodyetia bifurcata fruit peel extract: multifaceted potential in wound healing, antimicrobial, antioxidant, and anticancer applications
Source: Front Pharmacol. 2024 Aug 5;15:1435222. doi: 10.3389/fphar.2024.1435222 (PMC11330823; doi:10.3389/fphar.2024.1435222)
Supplement: Supplementary file 1 [file Table1.docx]

**Supplementary File**

### **Green Synthesis of Zinc Oxide Nanoparticles from** ***Wodyetia bifurcata* Fruit Peel Extract: Multifaceted Potential in Wound Healing, Antimicrobial, Antioxidant, and Anticancer Applications**

**Table S1.** FTIR analysis of ZnONPs depicting the different functional groups

| **Wavenumber (cm⁻¹)** | **Functional Group** |
| --- | --- |
| 3388 | O-H stretching |
| 2985 | C-H stretching (asymmetric) |
| 2927 | C-H stretching (symmetric) |
| 1638 | C=O stretching (amide I) |
| 1555 | N-H bending (amide II) |
| 1397 | C-H bending (methyl group) |
| 1013 | C-O stretching (alcohols, ethe |
| 810 | Zn-O stretching |
| 641 | Zn-O bending |

**Table S2.** Crystalline Size from XRD data by Scherrer Equation:

| **2 θ °** | **FWHM, °** | **Crystalline size D(mm)** |
| --- | --- | --- |
| 16.95128 | 0.173 | 46.42780154 |
| 29.67456 | 0.11 | 74.71193092 |
| 31.74246 | 0.3613 | 22.85950083 |
| 34.38211 | 0.2458 | 33.83157634 |
| 36.22277 | 0.3625 | 23.05769717 |
| 47.52602 | 0.5162 | 16.81556758 |
| 56.551 | 0.4271 | 21.12065256 |
| 62.74129 | 0.5052 | 18.41732621 |
| 66.45771 | 0.5249 | 18.09333048 |
| 67.98882 | 0.5472 | 17.51080679 |
| 69.11384 | 0.4523 | 21.32708859 |
| 72.49519 | 0.3593 | 27.41636743 |
| 76.84406 | 0.7064 | 14.3545812 |
| 81.46261 | 0.6475 | 16.19098643 |
|  |  | Average=26.58109 |

**Table S3.** Phytochemical analysis of *W. bifurcata* fruit peel extract.

| **S. No** | **Tests** | **Results** |
| --- | --- | --- |
|  | Alkaloids | - |
|  | Flavonoids | + |
|  | Saponins | + |
|  | Steroids | + |
|  | Triterpenoids | + |
|  | Tannins | - |
|  | Phenols | - |
|  | Glycosides | - |
|  | Anthraquinones | - |
|  | Coumarins | - |
|  | Diterpenes | - |
|  | Catechin | - |
|  | Anthocyanosides | - |
|  | Resins | + |
|  | Volatile Oil | - |

*“+” indicate positive results and “–” indicate negative results.*

**Table S4.** UV absorption showing the effect of temperature

| **S. No** | **Temperature (℃)** | **Peak value of the spectrum (nm)** | **Absorbance** |
| --- | --- | --- | --- |
|  | 30 | 364 | 0.7439 |
|  | 40 | 358 | 0.2850 |
|  | 60 | 354 | 0.2489 |

**Table S5.** UV absorption showing the effect of pH

| **S. No** | **pH** | **Peak value of the spectrum** | **Absorbance** |
| --- | --- | --- | --- |
|  | 6 | - | - |
|  | 8 | 358 | 0.4083 |
|  | 11 | 264 | 0.3037 |

**Table S6.** Antifungal activity against *C. albicans.*

| **Fungal strains** | **Antifungal Standard Fluconazole (mm)** | **ZnONPs (mm)** | **(FT) Extract (mm)** | **FT + Synthesised ZnONPs (mm)** |
| --- | --- | --- | --- | --- |
| *C. albicans (Lab strain)* | 27 ±0.272 | 9 ±0.136 | - | 12 ±0 |
